# Mothering in the streets: Familial adaptation strategies of street‐identified Black American mothers

**DOI:** 10.1111/jomf.12848

**Published:** 2022-05-18

**Authors:** Brooklynn K. Hitchens, Ann M. Aviles, Kathleen McCallops

**Affiliations:** ^1^ Department of Criminology and Criminal Justice University of Maryland College Park Maryland USA; ^2^ Department of Human Development and Family Sciences University of Delaware Newark Delaware USA

**Keywords:** community participation/action research, criminal justice system, family stress, inequalities, motherhood, resilience

## Abstract

**Objective:**

Using components of the Family Adjustment and Adaptation Response Model, Critical Race Feminism, and Sites of Resilience, this study explored how street‐identified Black American mothers engage in street life, while juggling the pressures of childrearing, family, and home life within a distressed, urban Black community.

**Background:**

Street‐identified Black American mothers are vilified for their intersecting identities of being Black women who are experiencing poverty, and who may also be involved in illegal activity. Black mothers are disproportionately represented in the criminal legal system, but existing research has inadequately examined how street‐identified Black mothers “do” family in the confines of structural violence.

**Method:**

We addressed this gap by analyzing interview data with 39 street‐identified Black American mothers ages 18 to 54. Data were collected using street participatory action research.

**Results:**

We identified a typology of three adaptive mothering strategies employed by street‐identified Black women as they respond to and cope with violent structural conditions shaping their mothering: constrained mothering, racialized mothering, and aspirational mothering.

**Conclusions:**

Findings suggested that these strategies were developed in response to an overarching carceral apparatus, of which these mothers were tasked with avoiding when possible and confronting when necessary. Their mothering strategies were shaped by a collective, Black American cultural identity and worldview, and the mothers possessed a unique way of perceiving the world as criminalized subjects with disproportionate proximity to the punitive state.

Low‐income Black American mothers who engage in *street life* do so while juggling the pressures of childrearing, family, and home life in the inner city (Hitchens & Payne, 2017, Forthcoming; Payne, 2008, 2011). We use the term *Black American* to identify American Descendants of Slavery and enslaved Africans. This specific racial classification underscores that Black Americans have a distinctive, cultural lived experience from broader groups and other Black people (e.g., Black African immigrants or Caribbean immigrants). “Street life,” “the streets,” or a “street” identity is phenomenological language that speaks to the various modes of survival in distressed, urban Black communities (Payne, 2008, 2011). These phenomenological concepts have been used by those in low‐income Black spaces to demarcate a subjective, cultural lived experience and worldview (e.g., “I'm from the streets” or “I come from a street family”). This conceptual worldview makes reference to oneself, family, or network in relation to a context of struggle and challenging or inequitable living conditions.

To cope with or manage stressors within this context, Black women and girls who are “street‐identified” embody a racial‐, ethnic‐, sociocultural‐, and gender‐based ideological code and behavior centered on attaining personal, social, and economic survival (Hitchens & Payne, 2017, Forthcoming; Payne, 2008, 2011). This blended system is often referred to as “grinding,” or the “underground” work that street‐identified Black women and girls employ to secure financial stability and also “reflects the intensity and drudgery of work in their daily lives” (McCurn, 2018, p. 8). For Black women and girls in the streets, bonding activities include interpersonal acts (e.g., attending parties or social events), group solidarity (e.g., participating in social clubs or “hanging on the block”), and community organizing (e.g., back‐to‐school drives or rent parties; Hitchens, 2020). Illegal activities include a diverse array of “hustles” that are generally stigmatized by mainstream society and can be nonviolent (e.g., drug use or sale, prostitution, or shoplifting) or violent (e.g., fighting, robbery, or gang involvement; Fishman, 1995; Jones, 2010; Maher & Daly, 1996; Miller, 1998). Some Black mothers, for example, navigate street life by employing situational forms of violence primarily to protect themselves and their loved ones (Hunt & Joe‐Laidler, 2001) or engaging in crime to supplement low‐wage work within the formal economy (McCurn, 2018). Still, the vast majority of Black women and girls do not participate in what is formally considered “crime,” even within low‐income or urban neighborhoods, and Black Americans are not a monolithic racial or ethnic group (Gaston & Doherty, 2018). Thus, street‐identified Black women and girls comprise a “rare” minority and hidden subset of the broader Black population (Heckathorn, 1997). Street life captures the fluidity of behaviors and activities of this subset, unraveling how they organize meaning around feeling well, satisfied, or accomplished and how they choose to survive through adversity (Hitchens & Payne, 2017; Payne, 2008).

When street‐identified Black American women and girls become mothers, they are tasked with the challenge of managing adversity while also navigating parenthood. We define Black American mothering as the various, context‐specific maternal strategies that Black American mothers employ to support their families under State violence and other oppressive forces (Collins, 1994; Dow, 2019; Gurusami, 2019; Rodriguez, 2016). Black Americans are often victims of *structural violence*, or the invisible, disproportionate, and excessive harm of the poor and marginalized as a function of their disadvantage (Gilligan, 1996). Structural violence describes how structural institutions and systems actively prevent individuals, groups, and communities from meeting their basic needs (Galtung, 1969). Gilligan (1996) argued that poverty is the deadliest form of violence and that social actors have a vested interest in the subjugation of Black Americans by preventing them from “realizing their potentialities” (Galtung, 1969, p. 170) through policies, laws, and other inequities.

This context of structural violence helped us to examine the conditions faced by street‐identified Black families and the complex demands placed upon Black mothers in the streets. Collins (1986) argued that being poor, Black, and female offers a “clearer view” of the “interlocking nature of oppression,” (p. 519), and anti‐Black racist misogyny is a form of discrimination particular to Black womanhood and produces racialized and gendered harm (Bailey & Trudy, 2018). Black mothers are not only disproportionately under correctional control relative to other mothers, but they also experience criminalization upon release that can constrain their post‐incarceration parenting practices (Gurusami, 2017, 2019; McCorkel, 2013). Formerly incarcerated Black mothers experience more difficulty regaining custody of their children upon release, despite the fact that they are typically primary caregivers prior to incarceration (Mitchell & Davis, 2019). Black mothers in the streets face hyper‐surveillance under conditions of probation or parole, and also navigate “family criminalization” or the intertwining institutional scrutiny and punitive treatment of Black mothers and their children (Elliott & Reid, 2019).

Our work demonstrates how this criminalization influences the “stigmatized identities” and racialized and gendered experiences of street‐identified Black American mothers (Moloney et al., 2011). We also reimagine how they resiliently traverse the terrains of mothering in low‐income, high‐crime neighborhoods. Examining the experiences of street‐identified Black American mothers contributes to the field of family science by highlighting how they engaged and fostered familial relationships, and particularly their role as mothers in the context of broader societal systems that are racialized, gendered, and classed. Furthermore, our work highlights the strengths of street‐identified Black American mothers, seeking to build upon their abilities to be self‐sufficient and resilient and to promote healthy family functioning in the face of adversity.

## STIGMATIZED IDENTITIES OF BLACK AMERICAN MOTHERS IN THE STREETS

Since the release of the Moynihan Report (Moynihan, 1965), Black families have been framed as a community in “crisis” and a “tangle of pathology” in academic and sociopolitical spheres; worse yet, the crisis has been perceived as self‐perpetuating. This report shaped the public lexicon about poor, single Black mothers as “the nation's newest boogeym[e]n,” “parasites,” “brood mares,” and “savage[s]” who could not be trusted to nurture their children or maintain a monogamous marriage (Orleck, 2005). Previous controlling images or stereotypes associated with Black women range from the mammy to the matriarch, the welfare queen, and the Jezebel (Collins, 1990). Friedman and Hitchens (2021) proposed that a new image, *The Criminal*, is a dominant schema that co‐produces and/or influences all other controlling images of Black women. The Criminal as a controlling image is enacted through criminal justice decision‐making and then inscribed onto the bodies of Black women and girls (Friedman & Hitchens, 2021). Anti‐Black rhetoric along with the advent of the War on Drugs solidified that the denigration of the Black family was owed, in large part, to the criminal negligence of poor Black mothers (Roberts, 1993).

Scholars on Black families have provided salient critiques of the culturally deviant models advanced in mainstream family literature (Burton et al., 2010; Few, 2007). These scholars noted that Moynihan's (1965) analysis was reflective of “white mainstream respectability” that placed blame on “absent” fathers and welfare “dependent” mothers (Orleck, 2005). Even more significantly, scholars demonstrated that poor Black mothers rarely see themselves through the racialized lens of Moynihan and others. For example, Orleck (2005) argued that single Black mothers resisted labels of being “unregenerate” and “burdensome” by white welfare caseworkers, and instead saw an “abundance of family values—not a lack of them” as instrumental in choosing to birth and raise their children (p. 84). Black mothers also nurture their children and cultivate community, while maintaining higher participation in the formal labor market than other mothers (McLoyd & Enchautegui‐de‐Jesús, 2005). The U.S. Bureau of Labor Statistics (2019) consistently finds that Black women ages 16 and older outpace other women in the workforce—an empirical finding that further debunks the myth of the lazy, “welfare queen” (Gilman, 2014). Burton et al. (2010) encouraged researchers in the field of family science to undertake more “robust understandings of racial socialization that move us toward truly understanding racialized systems effects in addition to race differences” (p. 455).

Many Black women have maintained individual and collective resilience even while they experience family provider role strain disproportionate to other women (Mendenhall et al., 2013). This resiliency is marked by ethnic‐specific strengths across the life course, including strong religiosity and extended family closeness (Mendenhall et al., 2013). Some scholars have continued to elucidate the intricacies of mothering in Black families, examining how parenting is a collective feature of the Black community (Gurusami, 2019). If children are not raised in a traditional two‐parent household, they are often raised in an extended family network of support in which kin and “fictive kin” help in child rearing (Collins, 1990).

Black American families are more complex and dynamic than is often captured in academic literature, especially for mothers in close proximity to the streets and/or criminal legal system. Street‐identified Black American mothers have conceptualized street life as a “site of resiliency,” or an adaptive way to secure opportunity for themselves and their families (Hitchens & Payne, 2017, Forthcoming; Payne, 2008). With this context in mind, we situated our qualitative findings in the family stress literature to examine how these mothers cope with the daily pressures of navigating family life while simultaneously enduring gendered racism, classism, and violent socioeconomic conditions. To better understand these interconnected familial and societal dynamics, we address the following research questions:

1. How do street‐identified Black American mothers make sense of the duality of street life and motherhood in the context of racial, economic, and gendered oppression?
2. How does this sense‐making inform street‐identified Black American mothers' parenting strategies when raising children in distressed contexts?
3. How do street‐identified Black American mothers demonstrate strength and resilience while parenting in these distressed contexts?

## THEORETICAL FRAMEWORKS

To understand the complex lived experiences of street‐identified Black American mothers, we employed critical theoretical frameworks on families that elucidate how adverse structural conditions influence their behaviors and parenting strategies. We drew on the Family Adaptation and Adjustment Response (FAAR) Model to situate this dynamic within a family system and understanding of family processes (Patterson, 2002). However, given the FAAR Model's racial and cultural limitations (Brown‐Baatjies et al., 2008), we expanded the Model with the addition of Critical Race Feminism (CRF; Wing, 1997) and Sites of Resilience (SOR; Payne, 2011). We employed CRF to create an intersectional lens of how multiple forms of oppression influence Black American mothering (Wing, 2000). We also utilized SOR to reconceptualize coping and notions of resilience, particularly the ideologies and behaviors considered “maladaptive” by the FAAR Model (Patterson, 2002; Payne, 2008, 2011). These theoretical additions provided support needed to understand the racial, ethnic, gender, and sociocultural worldviews of this stigmatized population of mothers who may be involved in illegal activities, fear confrontation with legal authorities, and/or experience various forms of economic or social precarity (Shaghaghi et al., 2011).

The FAAR Model constructs family resilience as the active process by which families adapt to risk exposure (Patterson, 2002). During the adjustment phase of the FAAR Model, families balance demands (i.e., stressors, strains, and hassles) that can co‐occur and accumulate over time, utilize capabilities (i.e., coping behaviors, resources, or support) to manage or alleviate demands, and find meaning (i.e., situational, family identity, and worldview) in how demands and capabilities shape their shared orientation (Patterson & Garwick, 1994). A family crisis occurs when demands on the family exceed their existing capabilities, creating an imbalance. During the adaptation phase, the family seeks to restore balance by reducing demands, acquiring new coping behaviors and resources, or changing their view of the crisis (Patterson, 1988).

While the FAAR Model is useful to identify how families grapple with the myriad crises that can disrupt households, it does so void of an intersectional analysis that recognizes that all crises are not made equally. CRF provides the latitude needed to center the marginalized experiences of street‐identified Black American mothers as individuals, while also contextualizing these experiences within the diverse Black family unit (Crenshaw, 1991; De Reus et al., 2005). Critical race feminists contend that there is no singular Black perspective or monolithic “Black experience,” and that theorizations on Black populations should include all that makes them rich and distinctive (Evans‐Winters & Esposito, 2010; Harris, 1990; Wing, 2000). Mothering in low‐income Black communities involves navigating a unique set of daily stressors and crises that emerge as a function of living in a “violent milieu” with multiple, interlocking forms of deprivation experienced at individual, group, and community levels (Jenkins, 2002, p. 30). As individuals, Black women continue to be violently victimized more frequently than other women, and this victimization often accrues over their lifetime (Sabri et al., 2016). Black women are murdered at higher rates than other women, and pregnant Black women are more than three times as likely to experience intimate partner homicide victimization as pregnant white and Hispanic women (Kivisto et al., 2021; Threadcraft & Miller, 2017). Black women are more likely to witness someone being shot, see a dead body, or hear about someone that they knew being killed or raped than other women (Isom Scott, 2018). CRF helped us to understand how these accumulated individual‐ and group‐based risks can influence Black mothering, while also shaping the adaptive well‐being of the entire Black communities.

The FAAR Model argues that “bonadaptation” (i.e., restoring balance) and “maladaptation” (i.e., poor adaptation) are two primary outcomes for families in crisis (McCubbin & Patterson, 1983; Patterson, 2002). As families adjust to accumulated demands, they rely on acquired resources and coping behaviors to address stressors (Patterson, 2002). Coping is traditionally theorized as “maladaptive” if the individuals or families do not effectively minimize stressors or engage in “avoidance” behaviors such as violence, drinking, or social isolation (Masten, 2001; McCubbin & Patterson, 1983; Scarpa & Haden, 2006). Yet, these traditional coping paradigms are insufficient to understand coping among Black populations, particularly in racially‐stressful situations (Plummer & Slane, 1996). Designed by and for white men and women, these paradigms often give value to Eurocentric ideals of self‐mastery, autonomy, and individualism while devaluing Afrocentric, collectivistic forms of coping which often include the local community (Banyard & Graham‐Bermann, 1993; Yeh et al., 2006). The model also reflects a relatively linear process in which families experience an imbalance, resulting in an adjustment, and then adaptation response to restore balance. However, the imbalance street‐identified Black American mothers face is constant due to the structural violence encountered within their communities and interactions with the State.

Moreover, the FAAR Model does not fully consider how nontraditional forms of coping are adaptive by fostering resilience in violent social conditions (Payne, 2011). SOR theory provided an alternative framing of how Black Americans engage in resilience within these conditions, by conceptualizing a street identity in terms of (a) phenomenology, (b) relational coping, (c) historical patterns/trends, (d) structural systems, and (e) incidents of social injustice (Payne, 2011). SOR argues that “criminal coping” is not only a major behavioral adaptation to chronic strain, but also that families who engage in street life do so to achieve restoration of the familial unit (Agnew, 2007; Payne, 2008, 2011). Street life becomes a “problem‐solving effort of the family system” (Brown‐Baatjies et al., 2008, p. 90) and street‐identified Black mothers who participate in illegal activities typically do so to confront the “strainful” effects of economic poverty and structural oppression (Agnew, 2007; Hitchens & Payne, 2017, Forthcoming; Payne, 2008, 2011). These conditions draw some Black women and girls to the viability of the streets and also increase their likelihood of using crime to achieve culturally defined goals and means of success (Agnew, 2007; Burt & Simons, 2015). Black mothers in the streets find psychological and physical spaces of resilience that operate concurrently to produce sites of strength at the individual, familial, and community levels (Hitchens, 2020; Hitchens & Payne, 2017; Payne, 2011). They manage chronic strain through bonding activities such as attending parties or frequenting bars, participating in social clubs such as motorcycle or car clubs, “hanging on the block” or street corner, and attending group gatherings with friends. They cultivate joy in times of sorrow by developing support groups for bereaved women and care for the children of mothers who fall on hard times (Hannays‐King et al., 2015; Hitchens, 2020). Survival and resilience are central to the Black American experience, and their traumatic history necessitates diverse forms of coping to combat their oppression (Sharpe, 2016).

## METHODS

We analyzed interview data from the Street Participatory Action Research (Street PAR) Health Project in Wilmington, DE. This project used a cross‐sectional, convergent mixed‐methods design to examine the relationship between health, structural opportunity, and violence among a large community sample of street‐identified Black Americans ages 16 to 54 in the Northside and Westside neighborhoods of Wilmington. These neighborhoods were selected as part of a multi‐neighborhood design focused on elevated rates of crime in the city of Wilmington. Data for this project were collected from April 2017 to October 2018, and the project lasted until December 2019.

The total project sample included 771 community‐based surveys, 72 in‐depth, semistructured interviews, and 22 months of ethnographic field observations collected by and on street‐identified Black men and women, consistent with the Street PAR approach. The project also implemented a robust “action” or activism agenda that ran concurrently with the empirical study. This manuscript solely examines the qualitative interview data from street‐identified Black American mothers ages 18 to 54 (*n* = 39).

### Street PAR

The convergent mixed‐methods design was situated within a Street PAR methodology. PAR is a methodological framework where formal researchers select members of the target population to mutually design and implement a research program while engaging in activism within a local community (Baum et al., 2006). PAR actively includes members of the “researched” in the research process, including developing research questions and collecting and analyzing data (Baum et al., 2006). As an application of PAR, Street PAR is an epistemological orientation that primarily involves street‐identified populations in schools, correctional facilities, and local communities to engage in this participatory enterprise (Payne, 2008). Street PAR assumes that individuals who are actively or formerly involved in the streets are best placed to systematically examine the sociostructural experiences of an urban and/or street population. As such, Street PAR embraces the worldviews and lived experiences of this population, shifting the location of power and knowledge production (Baum et al., 2006).

### Research site

Wilmington is the largest city in Delaware, and at the time of data collection (2017–2018), it had approximately 70,166 residents with 58% of the population Black and 36% of the population white (U.S. Census Bureau, 2019). Wilmington is a useful research site to examine the nexus of street life and mothering as the city has one of the highest per capita homicide rates for a city of its size (Federal Bureau of Investigation [FBI] Uniform Crime Reporting Program, 2009–2018; Jones, 2014). Labeled “MurderTown USA” and one of the “most dangerous small cities in America” (Jones, 2014), Wilmington has a violence problem that costs Delaware $611 million per year (Giffords Law Center, 2018).

### Participant recruitment

Participants had to meet the following criteria: (1) self‐identify as Black or African American; (2) self‐identify as female; (3) live in a low‐income environment; (4) be between the ages of 16 to 54; (5) live in the Westside or Northside neighborhoods of Wilmington; and (6) be street‐identified, involved with crime, formerly incarcerated, or formerly involved in activities associated with the streets. Participants were recruited using snowball sampling, a technique generally used to collect data from fringe, sparse, and/or sensitive populations (Shaghaghi et al., 2011). Participants who met the inclusion criteria were identified through the Street PAR Associates' social networks. The Associates were 11 street‐identified Black men who lived in Wilmington and were members of a previous Street PAR project. The Associates provided contact information for those who met the study criteria. The first author (Co‐Project Director) and second author (Co‐Investigator) reached out to interview the girls and women who the Associates identified and expanded the sample by asking those who participated if they knew additional individuals who fit the criteria.

### Interview subsample

A total of 50 street‐identified Black American girls and women were interviewed, of which 39 (78%) identified as mothers. At the time of the interview, 20 were custodial mothers (i.e., always had custody from birth), 2 were noncustodial mothers (i.e., never had custody from birth), and 17 had periods throughout their lives of being custodial and noncustodial mothers. Twelve of the women were also grandmothers (see Table 1).

**TABLE 1:** Characteristics of sample of street‐identified Black American mothers (*n* = 39)

Demographic characteristics	*N*
Age	
16–24	4
25–34	12
35–44	11
45–54	12
Employed	
Yes	17
No	22
Characteristics of mothers	
Custodial	20
Noncustodial	2
Periods of both custodial and noncustodial	17
Grandmother	12
Ever incarcerated	
Yes	12
No	27
Street involvement^a^	
Drug use	15
Drug sale	7
Sex work	6
Fighting/assault	39
Theft/robbery	4
Guns (around or use)	9

^a^ Some women engaged in multiple forms of illegal activity.

### Data collection

The in‐depth, semi‐structured interviews were designed to understand how the girls and women experienced the stress of violent crime in Wilmington, along with perceptions of, and attitudes toward, social institutions and state agents. Interviews were conducted primarily at community‐based centers in Wilmington as well as in participants' homes, workplaces, and other spaces based on their preferences. Most interviews were conducted by the first and second authors, along with a Street PAR Associate, Project PI, or graduate student. Participants provided their assent or consent to participate in the study by completing informed consent, video release forms, and a general demographic questionnaire. Participants received a $25 cash payment or gift card for the completion of an interview. Interviews typically took one to two‐and‐a‐half hours to complete. All participants were covered by a Certificate of Confidentiality. They were informed that participation was strictly voluntary and that all responses would remain confidential. All interviews were recorded using both audio and video recording equipment to ensure accuracy and later transcribed using a transcription service.

### Data analysis

For this analysis, we drew from a variety of interview questions such as: Can Black women be in the streets but still be a provider or protector? We ensured that the themes developed typified the most common patterns in the mother's accounts of their experiences and perspectives. These themes included resilience, motherhood, parenting strategies, community conditions, and family composition and structure. Grounded theory methods allowed for our data collection and analysis to inform one another through an iterative process of collecting, coding, comparing, reflecting, and creating themes (Charmaz, 2011). We developed a social justice inquiry by rejecting objectivity, emphasizing reflexivity, and developing themes that were sensitive to the nexus of power, privilege, and oppression (Charmaz, 2011). Coding proceeded in multiple stages to reduce the data into manageable, meaningful categories. At each stage, transcribed text was constantly compared and linked within and across interviews to develop meaning and relevance. Coding began with open coding, which included listening to and reading transcripts, coding the data line by line, selecting meaningful segments of the text, and labeling these segments into codes. We each open coded individually and then we met to discuss broad codes before moving forward. Focused coding followed and included further analyzing the codes derived from open coding. Focused coding also involved carefully reducing the number of codes by merging relevant codes and deleting codes that were not germane to the study. Followed by axial coding, we constructed linkages between codes to develop conceptual themes.

## FINDINGS

Black mothering among women in the streets involves a tightrope of stress and resilience. On this tenuous ground, street‐identified mothers use inventive strategies to juggle the precarity of living in violent social conditions while raising children, families, and communities. This precarity is most visible when examining how they negotiate multiple familial stressors in distressed ecological contexts with limited resources at their disposal. Street‐identified Black American mothers rationalize the duality of street life and motherhood by conceptualizing their street involvement in terms of survival—a mechanism to minimize, cope with, or alleviate complex forms of oppression. They learn to adapt to adverse conditions using a creative repertoire or “tool kit” that is shaped by their cultural habits, skills, and styles structuring their choices, behaviors, and decision‐making (Burt & Simons, 2015; Swidler, 1986). This repertoire includes using three types of fluid mothering strategies: (1) constrained mothering, (2) racialized mothering, and (3) aspirational mothering. Their mothering strategies are shaped by a collective, Black American cultural identity and worldview with a unique way of perceiving the world as criminalized subjects with disproportionate proximity to the punitive State.

### Constrained mothering: Economic deprivation in the streets

Black American mothers in the streets are compelled to contend with a myriad of stressors that complicate mothering, including living within a context of economic deprivation. Constrained mothering denotes how street‐identified Black American mothers navigated the margins of blocked socioeconomic opportunity by recalibrating their parenting strategies given the limited resources jeopardizing their ability to provide for and nurture their families. This context of economic hardship is emblematic of structural violence and can also be understood as “absolute” poverty or disadvantage, wherein individuals, families, or communities do not have the minimal level of resources needed for basic survival (Ladin, 2014). The mothers in our sample knew these hardships intimately, as most were either unemployed (*n* = 16) or were working in the low‐wage, part‐time sector (*n* = 15), and most lived in low‐income, multi‐family, or unstable housing (*n* = 24). While the women did not explicitly name policies enforced upon them, they did identify how various social policies (e.g., welfare or housing) served as stressors that shaped their mothering and contributed to their street involvement. Street‐identified Black American mothers rationalized the duality of street life and motherhood through an iterative assessment of individual and familial needs against the risks associated with satisfying those needs.

Housing insecurity frequently surfaced as a family stressor that contributed to the mother's use of constrained mothering to achieve stability for their families. Women spoke about living in homes where the “refrigerator is empty,” the “stove is messed up,” and living with “mice,” “roaches,” and other pests. Other women discussed crowded dynamics of “15 people” (Marshieka, 24) cramped into small apartments or street‐identified youth who “might be sleeping on the floor, air mattress, or whatever” (Kayla, 28). Evelyn (42) admitted that much of these “blended” family dynamics exist because poor families simply “can't afford to live alone.” And she's right—up until 2019, Delaware's minimum wage was $8.75, meaning a renter needed to work “100 hours, or 2.5 full time jobs, to afford the Delaware Fair Market Rent of $1,142 for a two‐bedroom unit” (Housing Alliance Delaware, 2019). Evelyn (42) revealed her own struggles with finding affordable housing for her and her children: “I haven't slept in my own bed since 2006. I've been on people's couches ever since.”

Some mothers countered these undesirable living arrangements by trying to “make their house a home” for their children. Dee (24) emphasized that, “I'm a mother and I have to make it home for my daughter. … You know, no matter where we are. We could… be in a box.” Other mothers instilled structure or routine in their children's lives through curfews, family dinners, and adequate clothing. Natasha B. (47) argued that even during her drug addiction, “I did everything for my kids. I made sure they had something to eat. … Clothes, roof over their head, lights. … When I was using [drugs], I made sure they was in bed, and they didn't see none of that.” Street life was seldom considered ideal or “good” behavior, but mothers who managed to balance both family life and their involvement in the streets were often granted a level of respect. According to Marquita (33), “if you involved in street life… it's not what you do, it's how you do it.” As a feature of constrained mothering, part of this balance included shielding their children from the more violent or dangerous sides of street life, including their own involvement. Kayla (28) contended:

[Mothers] might still be in the streets and they're kids be at school and [the kids] don't know … what [their mom is] into. So, I wouldn't necessarily say because a woman is in the streets that she can't be a great mom at home. … Because that might be her only means of being able to provide, depending on her background.

The mothers' second‐class positioning within a stratified labor market affected how they perceived their marginalized status and participation in crime (Crutchfield, 2014). Constrained mothering emerged as they saw involvement in illegal activity as a practical response to economic deprivation, given the lack of opportunity to attain livable wages within the labor market. Joy (37) explained how economic deprivation affects entire generations of families: “If you're raised in poverty, you don't have the income. … mom don't have money, family don't have money. So, you go to whatever you can do on the street… selling whatever you can sell just to make it.” Basheera (46) likened the desperate confines of poverty experienced by low‐income Black Americans as “trapping an animal in a corner. Eventually, the animal gonna come out fighting, doing whatever it needs to do to get where it needs to be.” Street life became a practical way to escape the vulnerability of concentrated disadvantage, and to provide basic needs for their families, even if stability was temporary or led to negative consequences for themselves or their children.

The practicability of street life was especially relevant if it meant providing food and sustenance for their children. On why Black women in the streets engaged in prostitution, Marshieka (24) rationalized that “maybe she doing it for her kids. … Maybe there is nobody that's helping her. … Ain't nobody prostituting for nothing. … Obviously, they providing for themselves or their kids.” Kayla (28) echoed this sentiment and believed that some poor Black mothers commit crime to address material needs of “feed[ing] their kids.” She said:

I feel like people engage in crime mainly for… survival needs. I think it starts there first with poverty. … A lot of people out here are struggling day‐to‐day to figure out how they're gonna feed their kids or figure out how bills is gonna get paid.

Several street‐identified mothers justified the use of constrained mothering to avoid literal or figurative hunger for themselves and their families. They demarcated their ability to “eat” or provide for themselves and their families as markers of survival. They grappled with the challenge of “do we eat or not eat” (Amanda, 29) or the literal fight to avoid hunger in bleak economic conditions, and often connected this fight to the viability of street life. Kimesha (22) made this connection seamlessly:

No, it's not that, it's ‘I need to eat today.’ If I don't get this money, if I don't sell this little ounce [drugs] in the next two days, for me, I'm not going to be able to eat. I'm not going to be able to pay the person that I'm staying with. … Backed up against the wall, you do what you got to do.

Kimesha (22) sold weed “just to have money in [her] pocket” after graduating and realizing that “nobody wanted to give [her] a chance.” Now as a mother to a newborn, she struggles to limit her involvement in street life while simultaneously hoping to instill “problem‐solving” strategies in her son's life: “Now that I got a little guy that I gotta teach how to be somebody's husband….I think the biggest thing I want to teach my son is like…you've got to know how to problem solve.” Kimesha (22) recognized the challenges that lie ahead for her son and the necessity of equipping her son with problem‐solving skills.

The sting of poverty left many street‐identified mothers feeling desperate at times, making difficult, real‐time decisions within the confines of their realities: “we make decisions that may not always be the best decision, but to us, it's presented as the only decision” (Amanda, 29). These choices could undoubtedly expose their families to harm within the carceral system, but were often seen as “worth it” given the pervasiveness of violent structural conditions in their lives. Tosha H. (47) underscored that poverty will test the limits of human dignity and survival, reflecting on when she would steal to not only feed her drug addiction, but also to stave off actual hunger:

We gonna steal because we gotta eat. What you want us to do? They cut them food stamps, all they gonna cause is destruction in our neighborhood. 'Cause you cut my food stamps, I'm comin' to your house. … It's gonna be like The Purge in the fucking 'hood. … So, like we gotta eat. And we will do whatever we got to do to survive. We are survivors. We outwit. We outplay. And we outlast. Period. … That's what we do here.

Tosha H. (47) described how engaging in street life (i.e., theft or violence) can be a site of resilience—a way to achieve personal, social, and economic survival (Payne, 2008, 2011). The demands of providing for their families in these constrained contexts, coupled with the capabilities available to the mothers, influenced their involvement in the streets to restore balance and survive—a function of their adaptation and adjustment.

Tosha H. (47) added that some enacted constrained mothering to navigate the margins of blocked opportunity by “flipping” welfare payments or food stamps, or by leveraging money or goods in hopes of increasing financial security. “Flipping” can include purchasing and selling drugs:

The $200 for your child [welfare direct payment]? What we gonna do for a whole month with $200? We gonna spend $50 a week? Huh? … it's not gonna go down like that. We gonna … take $150, go get this pack [drugs]. … $200 [will] be a thousand [dollars], before the whole month is out.

Black mothers in the streets also utilized constrained mothering to circumnavigate the same welfare regulations that increased their vulnerability to economic deprivation. At the time of her interview, Donielle (24), was experiencing homelessness and lived in her car. She highlighted flaws in welfare benefits that still do not meet the needs of impoverished families. She explained how many poor women sell their food stamps for practical reasons:

Baby needs Pampers. … phone bill needs to be paid. … and you've got these corner stores out here and you can't buy hot food. So, some people really sell a couple dollars of a food stamp, just to go buy some hot food.

Donielle (24) further underscored how the State used a “use‐it‐or‐lose‐it system” and did not allow food stamps to accumulate or roll over:

I had the stamps and my freezer broke. So by this time next month. … they just gonna wipe them stamps out. … They're not gonna double it. … I have to go hurry and sell every last one [or] go in there and use every food stamp 'cause they don't roll over.

Constrained mothering demonstrates the adaptive and inventive strategies street‐identified Black American mothers used to provide for their families while concurrently managing an enduring context of economic deprivation. Their mothering practices were also shaped by an embodied carcerality (Friedman & Hitchens, 2021) or the production of racialized, carceral subjects through reifying one's physical representation as synonymous with a deviant object. These situational and contextual constraints also shaped their coping behaviors. In the next section, we illuminate how racialized mothering is enacted in response to the criminalization of Black families, disproportionately through contact with an oppressive carceral apparatus.

### Racialized mothering: Criminalization in the streets

Racialized mothering refers to how street‐identified Black American mothers reconciled their familial relationship to the carceral apparatus as criminalized subjects and their adaptation to the racial dynamics embedded in their interactions with the criminal legal system. Race and racism continued to be central organizing principles that not only shaped the life chances of these mothers, but also structured their parenting practices and decision‐making. They were called on to raise grandchildren when drug addiction removed their daughters from the home, sent money on the books of their child's father in prison, and prayed for their sons who were shot while hanging on the block. Moreover, some of the mothers (*n* = 12) spent time in prison themselves, and half of the mothers (*n* = 19) experienced periods of noncustodial parenting due to incarceration, drug addiction, or other forms of street life. These multiple, overlapping identities can be understood as the “afterlives of slavery” or the continued suffering through truncated quality of life that is unique to the experiences of Black American descendants of the Slave South (Sharpe, 2016). Street‐identified Black American mothers shouldered the uneven burdens of care and emotional trauma as they not only felt the immense toll of mass incarceration when their loved ones were sentenced to prison, but also navigated their own involvement as criminalized subjects (Hitchens, 2020; Hitchens & Payne, Forthcoming). Racialized mothering revealed their strategies to survive the stress of the carceral state and the everyday state‐sanctioned violence pervasive in their communities, anchored by the intersections of race, ethnicity, class, and gender.

Seventeen of the mothers spoke of their experiences with either mothering children while incarcerated, mothering children who were incarcerated, or mothering children entangled in the carceral apparatus as juveniles on probation or having frequent police contact. Marshieka (24) and Martai (32) were both incarcerated in Baylor Women's Correctional Institution (BWCI) in Delaware for assault and drug possession, respectively. BWCI is a small women's prison that houses both pretrial detainees and those serving sentences, and most serve less than 1‐year sentences for nonviolent crimes (Delaware Department of Corrections, 2019). Both mothers illustrated the revolving door of incarceration among Black women from their neighborhoods. Marshieka (24) was incarcerated with her aunt who “[has been to jail] plenty of times. … we was in jail together, she got out, and she went right back in. She's in there now.” Martai (32) said both her stints in BWCI resembled a “family reunion”:

I was coming in and my sister was coming out. So, when my sister saw me, she was like, ‘What are you doing here?’ ‘Selling drugs again.’ So, then I was in there with cousins, like a family reunion. … Me and my family, the majority of us, was in there. … My sister did six months. … My cousin did three years. … My other sister, she didn't last that long, she got out. My oldest sister never been in jail.

The formerly incarcerated mothers interviewed acknowledged the ubiquity of the criminal legal system in their lives. Racialized mothering emerged as they sought to preserve their dignity through various actions, sometimes through garnering support and solidarity with other detained women and other times through acts of resistance (e.g., lashing out at prison guards). Their forms of adjustment and adaptation varied and were often shaped by the context, situation, and frequency of the challenges faced.

Entanglement within the carceral state apparatus also had a negative familial impact on many of the mothers. Lakeira (23) shared these difficulties:

A lot of the females, their kids was with family members, while there was other women whose kids was in the State. … I've seen it break a lot of women as well. I've been in the room with women who cried and [were] going through a DFS [Department of Family Services] situation, getting letters from DFS, like, ‘your kids is with the State because of incarceration.’ They would read a letter and just be like, ‘damn, my kids is getting took from me,’ and they'll break down crying.

As illuminated by Lakeira (23), mothering while incarcerated took a toll on many mothers who were separated from their children and given little to no control over their custody or placement.

In moments of vulnerability, formerly incarcerated mothers revealed how difficult it was to manage the lives of their children while “doing their time” in a system that felt cruel and unforgiving (Garcia‐Hallett, 2019). Lisa H. (37) lamented that being sentenced for robbery was “hell … because [she] missed [her] babies.” Lakeira (23) was forced to leave behind two young sons and remembered anxiously wondering about their well‐being: “Do my kids really remember me? Because you know, kids, they start to forget after a while. How are my kids? Are they being treated good? Are they eating enough? Are they bathed?” Lakeira was forced to wonder about her children as she described the prison environment where “they had us locked in a room for some petty stuff,” leaving her unable to call and talk to her children. When she tried other forms of communication, like sending letters, her letters were often never sent to her children, but rather read by prison guards and returned back to her due to what she had written to them. She said: “I used to be so over it. It's not somewhere anybody want to be. … I was being strong about it for a minute, but I actually got to a point where I had to physically fight somebody in there.” Martai (32) actually gave birth to her son while in prison and remembered being shackled during labor, with a leering guard at her side. She said she will never forget the trauma of being forced to leave her newborn at the hospital and said she began to lash out aggressively in prison:

Leaving? That was hard. That's when I just started tripping out in Baylor's. So, I stayed on lock[down] a lot of time. … Just not caring about nothin' and just, where my mental was at, all I wanted was my son. And then when you having visits when [your kids] come see you, and they don't wanna leave you, you don't wanna leave them, you just start going crazy. Like, you don't care about nothin' or nobody. You bucking, you're not listening to the guards. … I don't give a fuck. Where am I going?

While some may view Martai's (32) behavior as “maladaptive,” we understand her acts of resistance as rational responses to a challenging situation of leaving her newborn coupled with little to no control to alleviate the strain. This particular group of mothers relied on the coping strategies available to them within an environment of hyper‐surveillance and control. For Martai (32), this meant expressing her frustration by “bucking” and not complying with the rules set forth by a racialized system that continually enacted harm on her familial unit.

The racialized, carceral experiences of mothers extend far beyond jails and prisons. Five mothers spoke explicitly about their experiences with parenting a child who was previously or currently incarcerated. As mothers grappled with their children's incarceration, their *double consciousness* or understanding of the racial realities of society reflected a continuous ebb and flow of hope and struggle. They struggled to manage not being able to protect their children from harm in and outside of prison, while also maintaining hope that upon returning home, their children could successfully navigate a world that often contributed to their harm. Although the women did not explicitly name the structural violence created by society's policies, laws, and norms, their descriptions elucidated how their children and families were negatively impacted. Atif (35) shared how she supported her incarcerated son:

I talk to him and I just hope. … The last time that he was home, he was trying to do right but it was too late. … Before when he was home, he graduated, [and] he was doing what he is supposed to do, it ‘hit’ [home], like there's no way he's coming back [to prison]. … I talk to him, he sound a whole lot different. Now he's talking about his future, before he wasn't talking about his future, 'cause he wasn't sure he had one.

Atif's (35) remarks reflected the complexity of parenting a Black child and grappling with how structural violence truncated their ability to dream and develop a future orientation. She worked to help her incarcerated son stay focused and positive while reckoning with her lack of control of the situation. In the next section, we consider the strategies that the mothers used to adjust their cognitive worldviews as a form of adaptation to stressors.

### Aspirational mothering: Coping and survival in the streets

Aspirational mothering refers to the cognitive and emotional forms of survival street‐identified Black American mothers employ to cope with acute and chronic stressors that threaten their families. Building from Yosso's (2005) identification of various forms of capital, namely, aspirational capital—or the ability to maintain hopes and dreams for the future, even in the face of real and perceived barriers—aspirational mothering is another strategy employed to counter, and/or restore balance under violent structural conditions. Aspirational mothering emerged as these mothers made sense of their intersectional standing in society by altering their cognitive responses to stressors. They adjusted their mindsets and how they mentally and emotionally dealt with stressors by maintaining their hopes, dreams, optimisms, and aspirations to be self‐reliant and independent for their children's well‐being and safety. They transmitted their capabilities to their children by embodying strength not only as mothers, but also as Black women. We argue that Black mothering under the State is—in and of itself—a site of resilience that requires malleability against these pressures and structural conditions. Furthermore, while the characteristics of resilience enacted by the mothers overlap with the previous typologies described, we provide a distinct category here to outline the cognitive and mental shifts that mother's use, a practical adjustment they made as they sought to restore balance for their families.

Of significance is that all 39 mothers interviewed reported losing a loved one to homicide, reflective of the violence that has pervaded their lives and the subsequent emotional strain they are subject to. Street‐identified Black American mothers exhibited tremendous strength, even when they did not actually feel mentally or physically strong, to keep fighting for their family's success. At the time of Basheera's (46) interview, it was less than 2 months after her 18‐year‐old son was murdered. She viewed the tragic and untimely loss of her youngest child and burying him as an ultimate expression of her strength and independence as a mother and Black woman:

To me, I have to be a strong Black woman, for me and my family. 'Cause I'm the one that buried my children. So, I have to take care of them, no matter if the dad is there or not. I'm the one that's head of household.

This form of loss amplified the mother's perceptions of significant crises as they managed to survive traumatic life events while continuing to care for their families.

When experiencing a crisis, many street‐identified mothers engaged in mental or cognitive shifts to adjust to these difficulties. Audrey (43) called these shifts her ability to “keep her head above water” in her efforts to provide for her children. These shifts demonstrated how the mothers adjusted their mindsets and mentally and emotionally dealt with crises and stressors. Jasmine (27) suffered from drug addiction, and after losing custody of her toddler, lived in a homeless shelter. Despite being unemployed and housing insecure, she still expressed optimism and contentment:

I'm all right. I am 100% okay. I'm not okay with the state of my life right now, but I know it will get better. It's not forever. I live in a homeless shelter. I'm away from my child. Life is not great. I'm still smiling. I'm alright. All my needs are met. I've got a roof over my head. I've got food in my belly.

Jaynelle (31) admitted that she “fe[lt] down” after putting in her 2 weeks' notice for her cashier's job at Dunkin' Donuts. She exclaimed how she “love[s] working,” but could no longer endure the racism that she was experiencing in this position. With limited jobs available for her with a criminal record and limited education, she wrestled with going back to stripping to provide for her family. However, she remained hopeful that she could find legal employment, declaring “I went from rock bottom and now I'm going up.” We interpreted these shifts as how the mothers adjusted their worldview of their circumstances (crisis) and crafted the mental fortitude needed to survive.

This mental fortitude also encompassed how Black mothers in the streets reclaimed their dignity and independence often lost in the stigma of poverty. Many of the women personified great pride in “doing it for themselves” or “being a strong Black woman” and not “relying on men” to provide for themselves or their children. Donielle (24) had recently lost her job at Day's Inn, but refused to depend on her child's father for income: “What's gonna happen [if] dude gets locked up? What am I gonna do to have to pay bills now?. … that's why … I like to go get my own with it.” Marquita (33) tied being a Black woman to her ability to be independent and support her family:

I think a Black woman… we are more nurturers. … We are supposed to be there for our children, our men or whoever. … I think that's what being a Black woman means. Like providing for your children, providing for yourself. Being uplifting, being independent. Independency goes a long way. Regardless, if you're in the streets or not.

Street‐identified Black mothers embodied independence through self‐sufficiency to stem the tide of structural violence which often removed their men from the homes and left them unemployed or trapped in low‐wage work. Their understanding or perceptions of various crises served as the adaptation needed to survive and restore familial balance.

Women also engaged in aspirational mothering by “making a way out of no way,” and making decisions that stretched themselves in the low‐wage labor market to provide for their families and to keep them safe, even when that meant juggling long hours, multiple jobs, and time away from their children. Rather than living in subsidized housing, Mahogany (32) worked full time and paid market rate rent to keep her children “out of harm's way” or out of disinvested, often violent neighborhoods. Mahogany described how she was aware of the income‐based housing resources available to her, but how she mentally weighed the costs of moving to Riverside (public housing project) or staying and paying full rent to maintain her children's safety:

I mean, it's the balance of moving to Riverside and dealing with all that is going on over there, the shootings and the drug dealing and all that, or staying somewhere and paying full rent, but to know every day that my child is safer here than we probably would be there.

Netta (30) came to her interview after being awake for over 24 hours. She worked the overnight shift at a warehouse so that she was present for her kids every morning before they left for school, and she also attended classes to become a medical assistant. She described her hectic daily life:

My shift is usually 9pm to 6am. … I might go in at 6, 7[pm], so I can get the overtime by the end of the week. … so when I get off at 6 in the morning, I linger around the job. … I get my first group of kids out. My oldest two gotta be out by 7:30am. The next group don't have to be out til 8[am]. Once I do that, I shower between that, get the other group ready, and we leave out the door. I drop them off, and I go to class. … I get out of class at 2:30pm, I might take a quick nap and then cook dinner. By the time dinner get done, I go pick them up. Sometimes I make their plate first. … Then I'm back out the door to go to work.

Even with the various stressors placed upon street‐identified Black American mothers, they strived to maintain both strength and optimism and passed these capabilities to their children. As mothers, they wanted to show their children that, even while living in violent structural conditions, they still maintained hope for a better future, would be there to take care of them, and remained hopeful that they could teach their children to still “enjoy life.” Lisa H. (37) stated:

I just try to set a good example for my children and show them strength, and to not give up. … That I'm not gonna give up and I'm going to continue to be a good mother to them and take care of them, to make sure they have the things they need, and some of their wants. … To try to help them to enjoy life. … Even though things are not always good, you know, still have fun.

Aspirational mothering demonstrates the mental and emotional coping strategies street‐identified Black American mothers used to maintain their hopes, dreams, and optimism as well as their aspirations to be self‐reliant and independent while managing barriers. Their mothering practices were not only in response to the family struggles they endured, but also encompassed the structural conditions (e.g., low‐wage work and lack of affordable housing) that consistently influenced and shaped their capacity to address family demands. Few women “folded” or gave up on their families, even during the most challenging times in their lives. Instead, these mothers scraped by to survive during difficult times, crafting creative coping strategies to tend to the lives and limbs of their children and families.

## DISCUSSION

This paper contributes to existing literature by advancing three typologies that street‐identified Black American mothers utilize—constrained mothering, racialized mothering, and aspirational mothering—as a creative repertoire to adapt to pervasive and violent structural conditions. These parenting strategies were cultivated within the confines of their realities (i.e., meanings, demands, and capabilities), as these mothers work to achieve personal and familial balance (i.e., adjustment and adaptation) despite these strained conditions. Their decisions were informed by their individual, familial, and community identities and worldviews. Our specific focus on Black American women who are descendants of the Slave South allowed us to center their racialized, gendered, and sociocultural lived experience. These women have a distinct relationship with the State and constantly traverse multiple forms of structural oppression, engaging in mothering practices despite the barriers faced.

While much of the criminological and familial research on Black mothers has focused on incarcerated and formerly incarcerated women, we expanded these samples by examining Black American mothers with various proximity to the criminal legal system. Most of the mothers we interviewed were never incarcerated (*n* = 27), but all had some involvement in the streets and experienced shifting interactions with state agents. As such, they were each rendered “criminals” by governing bodies and institutions, given their marginalized identities. This marginalization shaped their parenting practices and decision‐making, including how they reconciled and/or worked to accommodate the duality of motherhood with street life. CRF and SOR theories provided the necessary underpinnings to expand the parameters of the FAAR Model by contextualizing this duality.

Our work illuminates the complex SOR embodied by mothers who access available capabilities such as physical aggression or mental toughness to survive in oppressive conditions, particularly when those conditions are considered unjust or high in magnitude (Agnew, 2007). It is through these frameworks that we complicate traditional understandings of families at the margins and unravel how street‐identified Black American mothers navigate the precarity of living in violent social conditions, seeking to achieve balance while raising and caring for children and families. The typologies described are generated as part of the mother's street identity and should not be understood as poor adaptive responses. Constrained, racialized, and aspirational mothering are strategies that support the mothers' capacities in balancing the continual demands imposed upon them by society. Moreover, the process street‐identified Black mothers engage in was nonlinear. In other words, their adjustment and adaptation were a constant ebb and flow of balancing demands and capabilities, shaping how they cope with adverse sociostructural conditions. Their street identity served as the anchor or common thread within each typology as they strived to meet the demands placed upon them.

The FAAR Model illuminated the daily hassles of navigating multiple stressors and challenges that affect families. CRF highlighted the racialized assumptions embedded in policies, specifically policies about Black “welfare queens” who abuse the system or buy “steak and lobster” with their food stamps rather than food to “stretch” for their children (Landsbaum, 2016). These rigid policies aid in keeping the mothers dependent on welfare assistance while also criminalizing their behaviors within that system—a system that is inherently racialized. The less popular truth is that many Black mothers in the streets use strategies (i.e., constrained and racialized mothering) as mechanisms to scrape by in a system not designed to meet their basic needs. Rightly or wrongly, the $1000 profit gained from illegally “flipping” welfare payments, for example, provides little financial security for these mothers. They grapple with this ongoing uncertainty, stretching themselves to the limits to provide for themselves and their families. The incorporation of SOR alongside FAAR and CRF acknowledges that Black mother's street identity serves as an adaptive mechanism of continuous adjustment in their efforts to restore balance in their lives and those of their families. When applied together, these models allowed for a more comprehensive examination of the processes by which street‐identified Black mothers seek to restore familial balance.

This study also has implications for how we theorize about the impact of surveillance and criminalization on Black mothering. As discussed by the mothers, too many women face the threat of not only incarceration, but also the fracturing of mother–child bonds. Roberts (2020) recognized that child removal from “protective” agencies (such as DFS) are an “integral part of the U.S. carceral regime” (p. 3). Removal often involved perceived neglect stemming from poverty, with punitive solutions that rarely addressed the structural causes of the problems Black families face (Gurusami, 2019). In overpoliced communities like Wilmington, low‐income Black mothers often contend with “nonnormative” events (Patterson, 1988) of violence and their children being incarcerated (Jones, 2014). While mothers tried to keep their children away from harm, they had to adjust and adapt by making sense of the “unexplainable” (Patterson, 2002). Street‐identified Black American mothers employed racialized mothering as a strategy to adjust to the racial dynamics embedded in their interactions with the carceral apparatus. Their use of racialized mothering demonstrates the labor needed to navigate motherhood as criminalized subjects. Black mothers in the streets also sought to restore the balance of their family demands by adjusting their perspective and outlook. Their use of aspirational mothering allowed them to hold a future orientation and hope for their children's reentry, while highlighting their sense‐making of the social forces their children must negotiate upon their return from prison.

Future scholarship might consider how to reframe dominant narratives of street‐identified Black American mothers and integrate frameworks that illustrate their strengths and mechanisms of resilience. Family theories such as the FAAR Model can be expanded to contextualize the racial, ethnic, sociocultural, and gendered experiences and worldviews of this population. Family scientists must not underestimate the power of racialized and gendered systems that sustain poor, inequitable, and violent socio‐structural conditions that continually harm Black mothers and their families. Furthermore, we must continue to complicate narratives of the “strong Black woman” archetype (Beauboeuf‐Lafontant, 2009). Just because women are able to demonstrate resilience in the face of extreme adversity does not mean it is a just burden. To quell the intersectional forms of violence perpetrated against Black mothers, structural interventions are needed, including fair housing wages, creating a child welfare system that does not function as an apparatus of the carceral state, and programs and policies that center the voices and experiential knowledge of street‐identified Black American mothers. Each of these interventions can alleviate the stressors that shape gendered and racialized pathways into street life for Black American mothers while enhancing their efforts to restore balance and provide for their families.

## CONFLICT OF INTEREST

We have no known conflict of interest to disclose.
